# Recent Advances in Gynaecological Care: Diagnostic Innovations, Therapeutic Strategies, and Patient Outcomes

**DOI:** 10.7759/cureus.115210

**Published:** 2026-08-26

**Authors:** Rucha Ganu, Devyani Misra, Saumya, Shreya Vijayasankar, Aditi Gheewala, Aditi Tyagi

**Affiliations:** 1 Department of Stree Rog and Prasutitantra, Dr. D.Y. Patil College of Ayurved and Research Center, Pimpri, IND; 2 Department of Obstetrics and Gynaecology, Dr. Ram Manohar Lohia Institute of Medical Sciences, Lucknow, IND; 3 Department of Obstetrics and Gynaecology, Government Medical College, Satna, Satna, IND; 4 Department of Obstetrics and Gynaecology, The Tamil Nadu Dr. M.G.R. Medical University, Chennai, IND; 5 Department of Medicine and Pharmacology, Sumandeep Homeopathic Medical College and Hospital, Sumandeep Vidyapeeth Deemed to be University, Vadodara, IND; 6 College of Allied and Healthcare Sciences, Teerthanker Mahaveer University, Moradabad, IND

**Keywords:** artificial intelligence, gynaecological care, molecular diagnostics, patient outcomes, targeted therapy

## Abstract

Gynaecological care has advanced substantially through innovations in diagnostic imaging, molecular profiling, minimally invasive surgery, fertility preservation, targeted therapies, digital health, and patient-centred care. Despite these developments, major challenges remain, including delayed detection of ovarian and endometrial malignancies, unequal access to advanced technologies, limited long-term validation of emerging interventions, and inadequate integration of quality-of-life outcomes into routine clinical practice. This narrative review aimed to synthesise recent progress in gynaecological care with emphasis on diagnostic innovations, therapeutic strategies, and their influence on patient outcomes. Relevant peer-reviewed literature published between 2018 and 2026 was reviewed using major scientific databases, including PubMed, Scopus, Web of Science, and Google Scholar. The literature indicates that high-resolution ultrasound, MRI, molecular diagnostics, and artificial intelligence are strengthening disease characterisation and risk stratification, while liquid-biopsy approaches remain under investigation for molecular monitoring and other potential clinical applications. Robotic-assisted surgery, fertility-preserving approaches, immunotherapy, PARP inhibitors, and anti-angiogenic therapies are expanding individualised treatment options. Telemedicine, mobile health applications, and patient-reported outcome measures are also improving accessibility, engagement, and holistic care assessment. These advances collectively support a transition toward precision-oriented and patient-responsive gynaecological care. However, high costs, infrastructure limitations, ethical and regulatory concerns, unequal access, and the need for further clinical validation continue to restrict widespread implementation. Continued validation, multidisciplinary collaboration, and equitable healthcare strategies are essential to improve gynaecological outcomes globally.

## Introduction and background

Gynaecological disorders affect women across the life course and contribute substantially to morbidity, impaired reproductive health, reduced quality of life, and healthcare burden worldwide [1]. These conditions are heterogeneous and may arise through interacting nutritional, hormonal, inflammatory, metabolic, microbial, and genetic mechanisms [2]. Gynaecological malignancies may additionally contribute to long-term functional morbidity, including pelvic-floor dysfunction and related quality-of-life impairment [3]. The multifactorial nature of benign gynaecological disorders further supports the need for integrated diagnostic and therapeutic approaches rather than isolated symptom management [4].

Advances in reproductive biology have improved understanding of inflammatory and microbial influences on gynaecological disease [5]. Alterations in the gut and genital-tract microbiomes have also been associated with inflammatory and immune pathways relevant to several gynaecological conditions [6]. Contemporary care additionally recognises sexual health and effective patient-clinician communication as important components of comprehensive management [7]. Communication strategies can facilitate identification of concerns that may otherwise remain unrecognised and support patient-centred care [8]. Reproductive-tract microbiota research continues to provide additional insight into interactions between microbial communities and reproductive health [9].

Progress in gynaecological oncology has shifted management toward more biologically informed and individualised approaches, particularly in ovarian cancer [10]. Multimodal treatment strategies combining established therapies with newer sensitising and targeted approaches have expanded therapeutic options in gynaecological malignancies [11]. Fertility preservation has also become increasingly important in cervical cancer management, particularly for appropriately selected women of reproductive age [12]. Sexual dysfunction and survivorship-related concerns require consideration alongside conventional oncological outcomes [13]. Emerging interventions such as platelet-rich plasma are also being investigated in reproductive and gynaecological practice, although their clinical roles require further validation [14].

Despite these developments, substantial unmet needs remain in early diagnosis, equitable access to specialised care, fertility-sensitive management, and integration of quality-of-life outcomes. Disability and sexual-health concerns may further influence physical, psychological, and social well-being and should be incorporated into patient-centred care pathways [15]. Common benign disorders such as uterine fibroids also demonstrate considerable variation in symptoms and clinical presentation across the reproductive life course, reinforcing the need for individualised assessment [16]. These challenges, together with advances in molecularly informed oncology, minimally invasive treatment, fertility preservation, digital health, and supportive care, highlight both the progress and remaining implementation gaps in contemporary gynaecological practice.

Objective of the review

This narrative review aims to critically evaluate recent advances in gynaecological care, with emphasis on diagnostic innovations, contemporary therapeutic strategies, and their influence on patient outcomes. It also examines persistent clinical limitations and identifies future priorities for improving personalised, accessible, and patient-centred gynaecological care.

Methodology

A narrative review with a structured literature-search approach was conducted to synthesise recent developments in gynaecological care, with emphasis on diagnostic, therapeutic, reproductive, oncological, digital-health, and patient-centred advances. Relevant peer-reviewed literature was identified through searches of PubMed, Scopus, Web of Science, and Google Scholar. Search terms included “gynaecological disorders,” “diagnostic innovations,” “minimally invasive surgery,” “reproductive health,” “gynecologic oncology,” “molecular diagnostics,” “targeted therapy,” “digital health,” and “patient outcomes.” Literature published between 2018 and 2026 was prioritised to capture contemporary developments, although earlier landmark studies were retained when necessary to provide context for established or practice-changing advances. Eligible sources included peer-reviewed clinical studies, systematic reviews, meta-analyses, consensus or guideline-based publications, and other directly relevant articles addressing recent innovations in diagnosis, treatment, or outcome assessment. Non-peer-reviewed material, conference abstracts without accessible full text, and publications outside the defined scope were excluded. Sources were selected according to their relevance to the major thematic categories of the review, with preference given to recent clinical evidence, major guidelines, and studies directly supporting the diagnostic or therapeutic advance being discussed. From the selected literature, information was extracted on the type of diagnostic or therapeutic advance, disease or clinical setting, principal clinical application, reported benefits or outcomes, limitations, and relevance to patient care. These findings were organised according to the predefined thematic sections of the review and synthesised narratively to identify major developments, clinical implications, and remaining evidence gaps. Because this article was designed as a narrative rather than a systematic review, formal Preferred Reporting Items for Systematic Reviews and Meta-Analyses (PRISMA)-based study selection, quantitative screening counts, and risk-of-bias assessment were not undertaken. Accordingly, the literature search was intended to provide a structured and clinically focused synthesis rather than an exhaustive or fully reproducible systematic evidence evaluation. No quantitative synthesis, meta-analysis, meta-regression, pooled effect estimates, or independent statistical analyses were performed because of heterogeneity across study designs, populations, interventions, and reported outcomes.

## Review

Advances in diagnostic imaging technologies

Advances in diagnostic imaging have improved the characterisation and evaluation of gynaecological disorders. High-resolution transvaginal ultrasound remains an important first-line imaging modality because it provides real-time assessment of pelvic anatomy and adnexal pathology [17]. Ultrasonography is also widely used in the assessment of uterine fibroids and other structural pelvic abnormalities [16]. Doppler imaging provides additional information regarding vascular patterns and may assist in characterising suspicious pelvic lesions [18].

Magnetic resonance imaging (MRI), including diffusion-weighted and contrast-enhanced techniques, provides high soft-tissue contrast and is particularly valuable for local tumour staging, lesion characterisation, and assessment of complex gynaecological disease [19]. Its greater anatomical detail can complement ultrasound when precise assessment of disease extent is required.

Artificial intelligence represents an emerging advance in gynaecological imaging, with machine-learning and deep-learning approaches being investigated for automated lesion detection, classification, segmentation, and risk stratification [20]. These systems may improve diagnostic consistency and assist clinical decision-making, although external validation, dataset quality, interpretability, regulatory oversight, and integration into routine workflows remain important limitations. Continued evaluation is required before widespread clinical implementation [20].

Molecular and genetic diagnostics

Molecular and genetic diagnostics have improved classification, prognostic stratification, and personalised treatment in gynaecological cancers [21]. In endometrial cancer, integration of molecular characteristics into contemporary classification and staging represents an important transition from predominantly clinicopathological assessment toward biologically informed risk stratification [21].

Endometrial cancer is increasingly classified according to The Cancer Genome Atlas (TCGA)-derived molecular groups: polymerase epsilon-mutated (POLE-mutated), mismatch repair-deficient (MMRd), p53-abnormal, and no specific molecular profile (NSMP) [22]. These molecular groups provide clinically relevant prognostic information and contribute to risk stratification and treatment planning. The Federation International of Gynecology and Obstetrics (FIGO) 2023 staging framework incorporates molecular characteristics into contemporary endometrial cancer classification [21]. MMRd status has additional clinical relevance for hereditary cancer assessment and molecularly informed therapeutic stratification [22].

Molecularly guided treatment has also become increasingly important in ovarian cancer. SOLO1 demonstrated the efficacy of maintenance olaparib in patients with newly diagnosed advanced BRCA-mutated ovarian cancer [23]. Beyond BRCA1/2 mutation status, homologous recombination deficiency (HRD) has emerged as an important biomarker for identifying additional molecularly selected patient groups [24]. PAOLA-1 demonstrated benefit from olaparib plus bevacizumab in HRD-positive advanced ovarian cancer, including HRD-positive disease without a BRCA mutation [24].

These developments illustrate the increasing role of molecular classification and biomarker-directed treatment selection in gynaecological oncology. Key contemporary diagnostic and molecular approaches are summarised in Table 1.

**Table 1:** Summary of Advanced Diagnostic and Molecular Technologies in Gynaecology MRI: Magnetic Resonance Imaging; DWI: Diffusion-Weighted Imaging; AI: Artificial Intelligence; TCGA: The Cancer Genome Atlas; FIGO: International Federation of Gynecology and Obstetrics; POLEmut: polymerase epsilon-mutated; MMRd: mismatch repair-deficient; p53abn: p53-abnormal; NSMP: no specific molecular profile; BRCA: breast cancer susceptibility gene; HRD: homologous recombination deficiency; PARP: poly(ADP-ribose) polymerase.

Technology/Modality	Application	Advantages	Limitations	References
Transvaginal Ultrasound	Pelvic organ assessment, ovarian cyst detection, fibroid evaluation	Non-invasive, cost-effective, real-time imaging	Operator-dependent and limited for detailed tissue characterisation	[16,17]
Doppler Imaging	Assessment of vascular patterns in suspicious pelvic lesions	Provides complementary information on tumour vascularity and lesion characterisation	Limited specificity in complex or indeterminate lesions	[18]
MRI (DWI, Contrast-enhanced)	Cancer staging, endometriosis evaluation, and soft-tissue characterisation	High soft-tissue resolution and detailed local assessment	Expensive and less accessible in resource-limited settings	[19]
AI-assisted Imaging	Automated detection, classification, segmentation, and risk stratification in gynaecological imaging	May improve diagnostic consistency and support clinical decision-making	Requires large validated datasets, external validation, interpretability, and regulatory oversight	[20]
TCGA-derived Molecular Classification and FIGO 2023 Staging	Molecular classification and integrated staging of endometrial carcinoma using POLEmut, MMRd, p53abn, and NSMP groups	Improves prognostic stratification and supports risk-adapted treatment planning	Requires validated molecular testing and specialised pathological interpretation	[21,22]
BRCA and Homologous Recombination Deficiency (HRD) Testing	Molecular stratification of ovarian cancer and identification of patients potentially eligible for PARP-inhibitor maintenance	Extends predictive assessment beyond BRCA1/2 mutations to selected HRD-positive, BRCA-wild-type tumours	Assay methodology and genomic-instability thresholds may vary	[23,24]

Minimally invasive and robotic-assisted surgery

Minimally invasive surgery has become an important component of contemporary gynaecological practice, offering reduced surgical trauma and faster postoperative recovery compared with open approaches in appropriately selected patients [25]. Laparoscopic techniques are widely used for procedures such as hysterectomy, myomectomy, and surgery for endometriosis, with potential advantages including reduced blood loss, shorter hospital stay, and faster recovery [25].

Robotic-assisted surgery provides three-dimensional visualisation, articulated instrumentation, and enhanced dexterity, which may facilitate technically demanding dissection and suturing [26]. These features may be particularly useful in complex gynaecological and oncological procedures requiring precise tissue handling [27].

Clinical evidence suggests that robotic surgery may facilitate minimally invasive completion of technically complex procedures and support operative precision [28]. However, cost-effectiveness compared with conventional laparoscopy remains uncertain because robotic systems involve substantial acquisition, maintenance, and procedural costs [28]. Access is consequently limited in many resource-constrained healthcare settings.

Despite these advantages, robotic surgery should be regarded as an additional minimally invasive surgical platform rather than a universally superior alternative to conventional laparoscopy. Appropriate patient selection, surgeon expertise, institutional resources, and procedure complexity remain important determinants of its clinical value [28].

Advances in reproductive medicine and fertility preservation

Advances in reproductive medicine have expanded options for fertility treatment and fertility preservation, particularly for individuals delaying childbearing and patients undergoing gonadotoxic cancer therapy [29]. Assisted reproductive technologies (ART), including in vitro fertilisation (IVF) and intracytoplasmic sperm injection (ICSI), continue to evolve alongside embryo-selection and fertility-preservation strategies. Time-lapse embryo imaging and preimplantation genetic testing may provide additional information for embryo assessment and genetic-risk evaluation in selected clinical settings [30].

Oocyte and embryo cryopreservation are established approaches to fertility preservation. Vitrification has improved the efficiency of gamete and embryo cryopreservation and supports long-term storage for reproductive use [30]. These approaches are particularly relevant for patients undergoing chemotherapy, radiotherapy, or other treatments that may impair reproductive potential [31].

Fertility preservation should be considered early in the management of reproductive-age patients with gynaecological malignancies [32]. Available strategies may include oocyte or embryo cryopreservation, ovarian tissue cryopreservation, and ovarian transposition depending on age, diagnosis, treatment urgency, and planned cancer therapy [32]. Timely counselling and multidisciplinary coordination between oncology and reproductive-medicine teams are therefore important components of care [32].

Despite these developments, cost, unequal access, specialised infrastructure, treatment-related urgency, and ethical considerations may limit availability of fertility-preservation services [32]. Continued research is needed to improve accessibility and optimise patient selection and long-term reproductive outcomes.

Targeted and personalised therapeutic strategies

Individualised treatment has become increasingly important in modern gynaecological practice, with therapeutic decisions increasingly informed by clinical characteristics and molecular profiles [33]. Hormonal therapies remain important in hormone-sensitive malignancies, uterine fibroids, and endometriosis, with treatment selection tailored to disease characteristics, symptom burden, and reproductive goals [34].

Targeted therapies have substantially expanded treatment options in gynaecological oncology. PARP inhibitors are particularly relevant in molecularly selected ovarian cancers, while anti-angiogenic therapies inhibit pathways involved in tumour vascularisation and progression [34]. Antibody-drug conjugates (ADCs) have also emerged as a biomarker-directed therapeutic approach in gynaecological oncology by combining tumour-targeting antibodies with cytotoxic agents. Their clinical use depends on appropriate tumour-target expression and patient selection [34]. More broadly, molecular profiling enables identification of potentially actionable alterations and supports selection of targeted therapies in appropriately characterised tumours [35].

Immunotherapy has become an important systemic treatment approach in selected advanced and recurrent gynaecological malignancies. Immune-checkpoint inhibitors enhance antitumour immunity by blocking inhibitory immune pathways, with treatment response influenced by tumour-specific biomarkers such as mismatch-repair status, microsatellite instability, and programmed death-ligand 1 (PD-L1) expression [34]. Integration of molecular biomarkers into therapeutic decision-making has therefore become increasingly important for identifying patients most likely to benefit from targeted and immune-based treatments [35].

Despite these advances, high treatment costs, unequal access, treatment-related toxicity, and acquired resistance remain important limitations. Future precision-oncology strategies are expected to increasingly integrate molecular profiling, biomarker expression, genomic characteristics, and emerging therapeutic targets to refine patient selection and treatment individualisation [36]. Therapeutic strategies and mechanisms are summarised in Table 2.

**Table 2:** Emerging Therapeutic Strategies in Gynaecological Care PARP: Poly(ADP-ribose) Polymerase; PCOS: Polycystic Ovary Syndrome; ADC: Antibody-Drug Conjugate; BRCA: Breast Cancer Susceptibility Gene; HRD: Homologous Recombination Deficiency.

Therapy Type	Mechanism of Action	Clinical Application	Advantages	Limitations	References
Hormonal Therapy	Modulates endocrine pathways	Endometriosis, PCOS, fibroids, and selected hormone-responsive conditions	Effective symptom control and individualised management	Adverse effects and limitations with prolonged use	[4,16]
PARP Inhibitors	Inhibition of DNA repair pathways	Molecularly selected ovarian cancers, particularly those with BRCA alterations or homologous recombination deficiency	Biomarker-directed treatment in selected patients	Acquired resistance and treatment-related toxicity	[34,36]
Antibody-Drug Conjugates (ADCs)	Tumour-targeting antibody delivers a cytotoxic payload	Biomarker-selected gynaecological malignancies expressing appropriate tumour-associated targets	Potential for more selective delivery of cytotoxic therapy	Target-dependent efficacy, toxicity, resistance, cost, and restricted patient eligibility	[34]
Immunotherapy	Immune-checkpoint blockade enhances antitumour immune responses	Selected advanced or recurrent cervical, endometrial, and other gynaecological cancers according to tumour biomarkers	Potential for durable responses in appropriately selected patients	Variable response, immune-related adverse events, and dependence on tumour biology and biomarkers	[34,35]
Anti-angiogenic Therapy	Inhibits tumour-associated blood-vessel formation	Selected advanced gynaecological malignancies	Provides a targeted approach to inhibition of tumour vascularisation and progression	High cost, treatment-related adverse effects, and resistance	[34,36]
Nanotechnology-based Drug Delivery	Nanocarrier-mediated targeted or controlled drug delivery	Primarily preclinical and early translational investigation in gynaecological oncology	Potential for improved drug targeting and controlled delivery	Predominantly preclinical evidence, with limited clinical validation and implementation	[36]

Management of gynaecological cancers

Management of gynaecological cancers increasingly requires disease-specific assessment incorporating tumour type, stage, histology, molecular characteristics, patient fitness, and treatment intent. Contemporary management may involve surgery, chemotherapy, radiotherapy, and targeted systemic approaches according to individual clinical circumstances [37].

Molecular and hereditary factors are also relevant to management in selected patients, particularly where inherited cancer-predisposition syndromes influence surveillance, counselling, and therapeutic planning [38]. Surgery remains an important component of treatment for many resectable gynaecological malignancies, with the extent and approach determined by tumour characteristics and treatment goals [39].

Earlier recognition of suspicious symptoms and appropriate referral remain important because delayed diagnosis can adversely affect treatment options and outcomes [40]. Despite therapeutic progress, recurrence, treatment resistance, unequal access to specialist services, and treatment-related morbidity remain important challenges. Global disparities in access to innovative diagnostic and therapeutic approaches may further limit the translation of advances in gynaecological oncology into equitable clinical outcomes [41]. Continued improvement in early diagnosis, personalised treatment, and equitable access to multidisciplinary care is therefore required.

Digital health and telemedicine in gynaecology

Digital health and telemedicine have expanded options for delivering gynaecological care, particularly by supporting remote consultation, follow-up, communication, and continuity of care [42]. Teleconsultation can reduce the need for in-person visits in appropriate circumstances and may improve access for patients living in rural or underserved areas [42]. Evidence from gynaecological practice indicates that telemedicine can provide a feasible alternative for selected consultations and follow-up encounters, although its suitability varies according to the clinical problem [43].

Mobile health applications and other digital platforms can support menstrual tracking, fertility monitoring, symptom reporting, and patient engagement [44]. Digital technologies may also facilitate collection and exchange of longitudinal health information, although their clinical value depends on appropriate validation and integration into healthcare systems [45].

Important barriers include data privacy, digital literacy, unequal access to technology, infrastructure limitations, and variable clinical validation [45]. Effective implementation therefore requires appropriate governance, secure data management, patient and clinician education, and consideration of disparities in digital access. The principal digital and patient-centred applications are summarised in Table 3.

**Table 3:** Digital and Patient-Centred Innovations in Gynaecology mHealth: Mobile Health; PROMs: Patient-Reported Outcome Measures; AI: Artificial Intelligence.

Innovation	Application	Benefits	Challenges	References
Telemedicine	Remote consultation and follow-up	Improves access and reduces travel burden	Requires digital infrastructure and adaptation by patients and clinicians	[42,43]
mHealth Applications	Menstrual tracking, fertility monitoring, and symptom self-reporting	Supports patient engagement and self-monitoring	Data privacy concerns and variable clinical validation	[44,45]
Wearable Devices	Physiological monitoring and longitudinal health tracking	Supports continuous monitoring and longitudinal data collection	Accuracy, variability, and limited integration into routine care	[45]
PROMs	Quality-of-life assessment and symptom monitoring	Supports holistic outcome assessment and patient-centred decision-making	Lack of standardisation and inconsistent routine use	[46,47]
AI-based Decision Support	Clinical prediction, risk stratification, and care planning	Supports diagnostic consistency and personalised decision-making	Ethical, regulatory, validation, and data-governance concerns	[20,45]

Patient-centred care and quality of life outcomes

Patient-centred outcome assessment complements the preceding discussion of digital health by focusing on aspects of care that may not be captured through conventional clinical endpoints. Patient-reported outcome measures (PROMs) provide structured information on pain, physical functioning, emotional well-being, sexual health, and overall quality of life that may not be captured by conventional clinical endpoints [46,47]. Their use can support longitudinal assessment and more individualised treatment decisions, particularly in chronic gynaecological conditions where symptom burden substantially affects daily functioning and quality of life [48].

**Figure 1:**
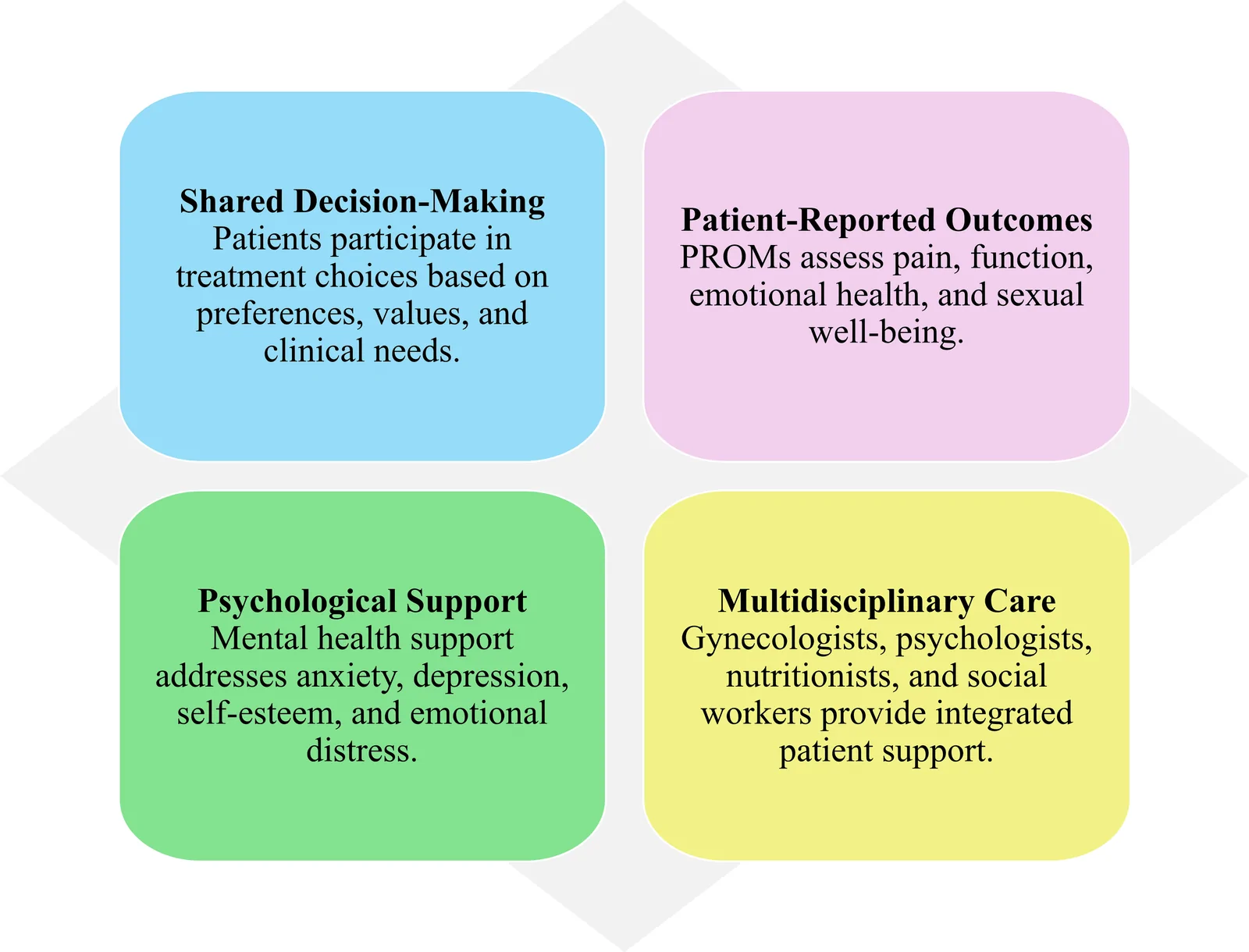
Patient-Centred Care in Gynaecology Created by authors using Microsoft PowerPoint (Redmond, WA, USA) based on information from references [1,7,8,13,46-48]. PROMs: Patient-Reported Outcome Measures

Psychological and supportive care are important components of comprehensive gynaecological oncology because psychological distress can adversely affect quality of life. Psychosocial interventions, including cognitive behavioural therapy, psychoeducation, and structured psychological support, may reduce psychological distress and improve psychological well-being and quality of life in women with gynaecological cancers [49]. Figure 1 summarises these patient-centred components.

Limitations and future directions

This narrative review has a few limitations. The included literature varies in study design, sample size, population, and reported outcomes, which limits direct comparison across studies. Because the review used a narrative rather than a systematic approach, source selection and interpretation may be subject to selection bias, and the absence of formal systematic screening and risk-of-bias assessment limits reproducibility and may have resulted in omission of relevant evidence. In addition, some emerging approaches, particularly AI-assisted diagnostics, liquid biopsy, nanotechnology-based drug delivery, and selected precision-oncology strategies, remain at different stages of clinical validation and should not be interpreted as uniformly established standards of care. Robotic surgery and molecular diagnostics may also be difficult to implement widely because of cost, infrastructure requirements, specialised expertise, and unequal access, particularly in resource-constrained settings. Data privacy, regulatory oversight, assay standardisation, and ethical governance remain additional concerns for digitally enabled and molecularly guided care.

Future research should prioritise large prospective multicentre studies, external validation of emerging diagnostic technologies, and longer-term assessment of clinically meaningful outcomes. Greater emphasis is also needed on affordability, equitable implementation, and integration of validated molecular, digital, and patient-reported measures into routine practice. Multidisciplinary collaboration and appropriately governed use of artificial intelligence may further support more precise and patient-centred gynaecological care.

## Conclusions

This narrative review highlights substantial advances in contemporary gynaecological care across diagnostic imaging, molecular classification, minimally invasive surgery, fertility preservation, targeted therapy, immunotherapy, digital health, and patient-centred outcome assessment. Improved imaging and molecular characterisation have strengthened disease stratification, while minimally invasive and robotic-assisted approaches have expanded surgical options in appropriately selected patients. Precision-oriented treatments, including molecularly guided systemic therapies, have further individualised the management of gynaecological malignancies, and advances in reproductive medicine and fertility preservation have broadened options for patients with reproductive goals. Digital health and telemedicine may also improve access, follow-up, and continuity of care. Despite these developments, major challenges remain, including unequal access, high costs, infrastructure requirements, treatment-related toxicity, and the need for further validation of emerging technologies. Future progress will depend on robust clinical validation, multidisciplinary collaboration, equitable implementation, and integration of patient-centred outcomes into routine practice. Overall, contemporary gynaecological care is moving toward a more precise, individualised, and accessible model, although widespread clinical benefit will depend on addressing these implementation barriers.
